# Supplementary material for: Risk and protective factors associated with brain grey matter patterns in a population-based cohort of cognitively unimpaired 70 years old
Source: BMC Med. 2025 Dec 17;24:39. doi: 10.1186/s12916-025-04583-0 (PMC12822138; doi:10.1186/s12916-025-04583-0)
Supplement: Supplementary file 1 — Additional file 1. Supplementary Figures and Tables (Figures S1–S4; Tables S1–S7).Fig. S1. Clustering output visualizations. (A) Two-dimensional MDS plot showing subject similarity based on cortical and subcortical grey matter (GM) patterns. (B) Hierarchical clustering dendrogram illustrating subtype grouping. Fig. S2. Five cortical thickness and subcortical volume GM patterns compared with the sample mean. Fig. S3. Cortical ROI rankings (Cohen’s d) identifying the most discriminative regions for differentiating clusters. Fig. S4. Baseline cortical thickness and subcortical volume values by cluster (averaged across hemispheres). Table S1. Test–retest reliability of FreeSurfer 7 cortical thickness and subcortical volume measures before/after scanner upgrade in two phantoms. Table S2. Baseline Alzheimer’s disease CSF biomarker positivity across clusters in 286 participants. Table S3. Comparison of baseline demographic, clinical, genetic, cardiovascular, and neuroimaging characteristics between participants with vs. without CSF data. Table S4. Multinomial regression models (clinical, neuroimaging, inflammation, CSF) reporting odds ratios for cluster membership relative to Cluster 1. Table S5. GLM results for associations between GM clusters and six cognitive outcomes at baseline. Table S6. Sex- and education-adjusted linear mixed-effects models for longitudinal trajectories of cognitive subdomains, MMSE, and WMHV. Table S7. Baseline sociodemographic, clinical, biochemical, neuroimaging variables, and GM clusters by survival status (follow-up, dropout, deceased). [file 12916_2025_4583_MOESM1_ESM.docx]

**SUPPLEMENTARY MATERIALS**

**Supplementary Figure 1.** Clustering output plots. **(A)** Two-dimensional multidimensional scaling (MDS) representation of the similarity matrix. Each dot represents a subject, with distances reflecting the similarity of their cortical and subcortical grey matter (GM) patterns. The axes correspond to the first and third MDS components of the random forest proximity matrix, reflecting overall amount of GM (Component 1) and regional heterogeneity), i.e. GM patterns (Component 3). **(B)** Hierarchical clustering dendrogram. The horizontal axis indicates the distance between subjects based on random forest similarity, while the vertical axis shows the hierarchical grouping structure. Colours represent different subtypes, as indicated in the legend.


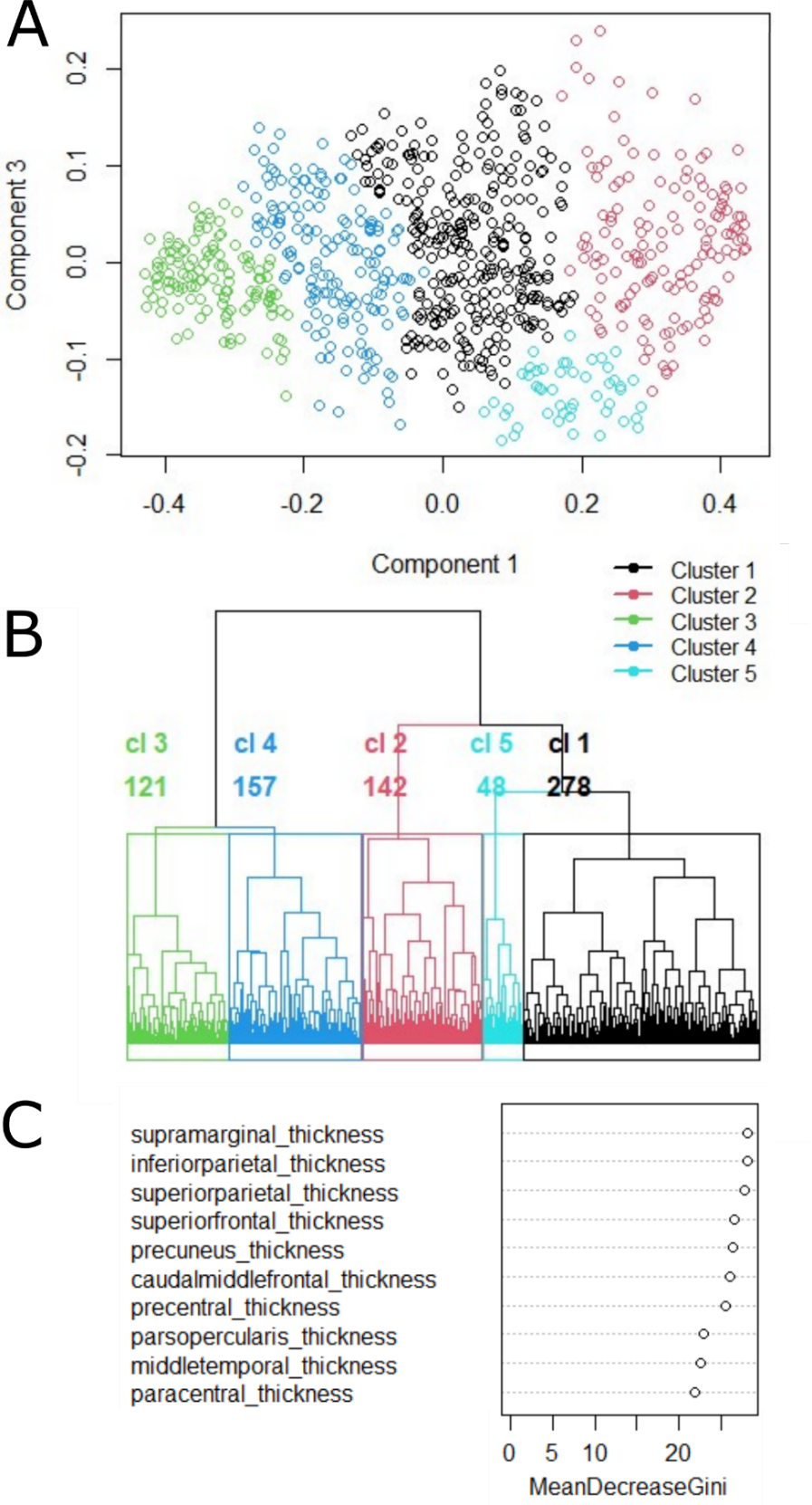


**Supplementary Figure 2.** Gray matter clusters. The five identified patterns of cortical thickness (left) and subcortical volume (right) compared to the sample mean.


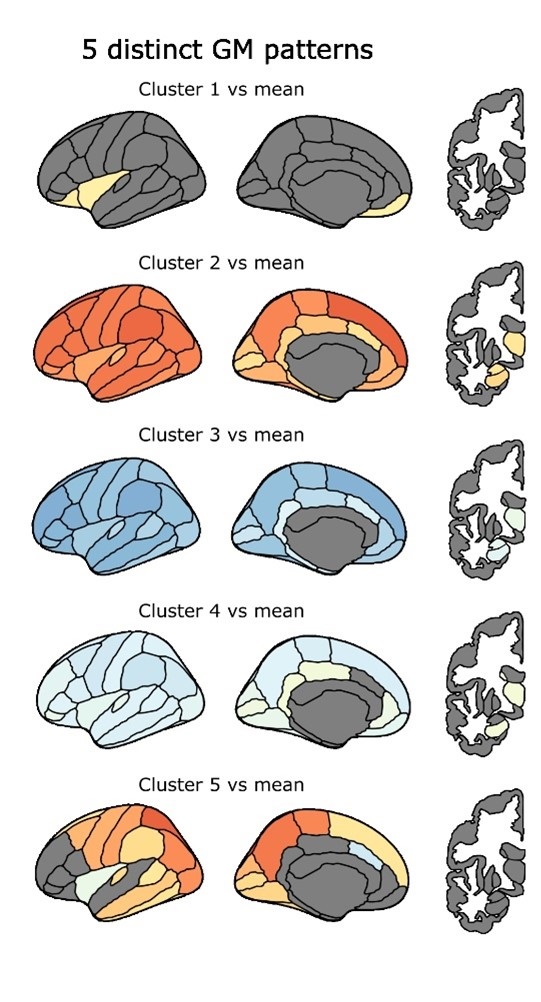

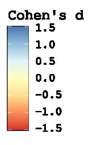


**Supplementary Figure 3**. Cortical ROI rankings based on Cohen’s d effect sizes (averaged across hemispheres) for pairwise comparisons between each cluster and Cluster 1. The plot highlights the three brain regions (ROIs) with the strongest discriminative power for differentiating participant clusters.

**
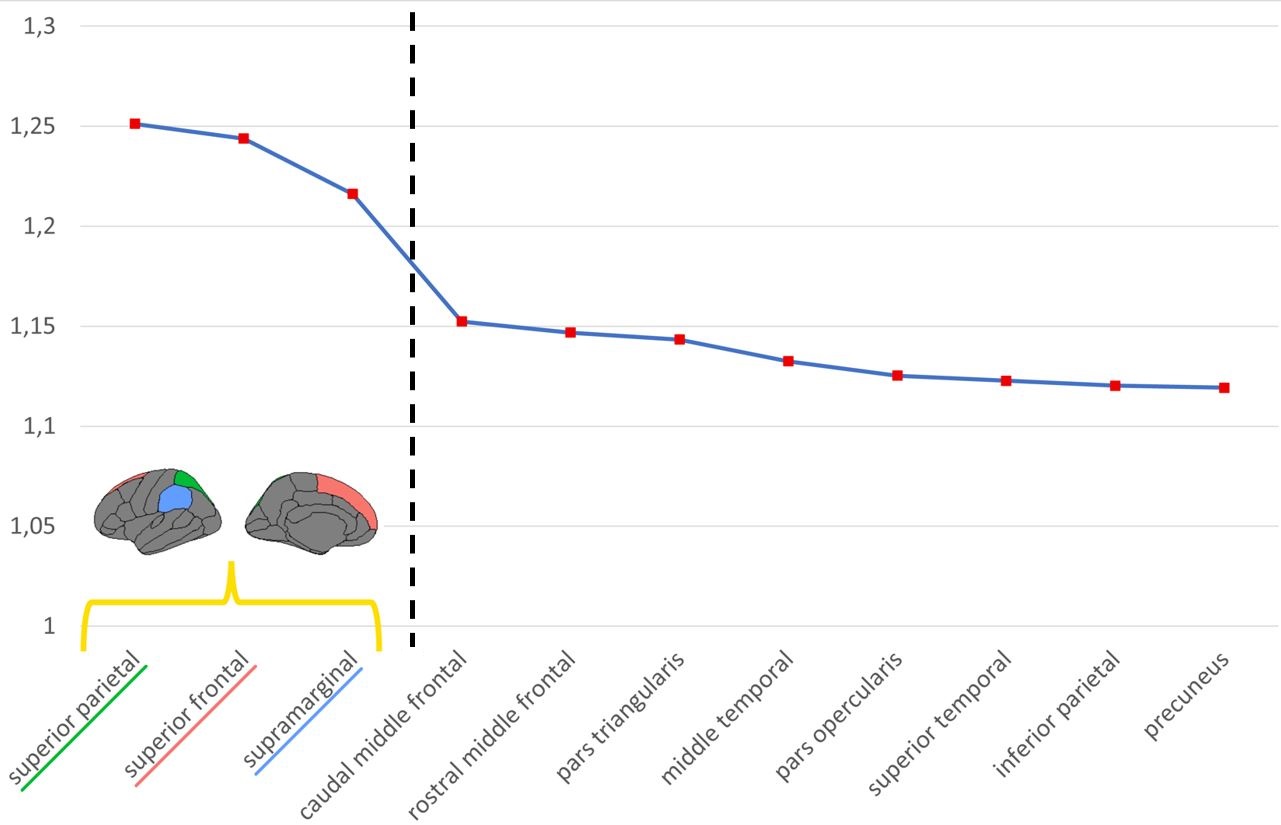
**

**Supplementary Figure 4**. Baseline brain ROI values by cluster. **(A)** Cortical thickness (mm) across all cortical regions. **(B)** Subcortical volumes (mm³) across all subcortical regions. All values are averages across hemispheres.


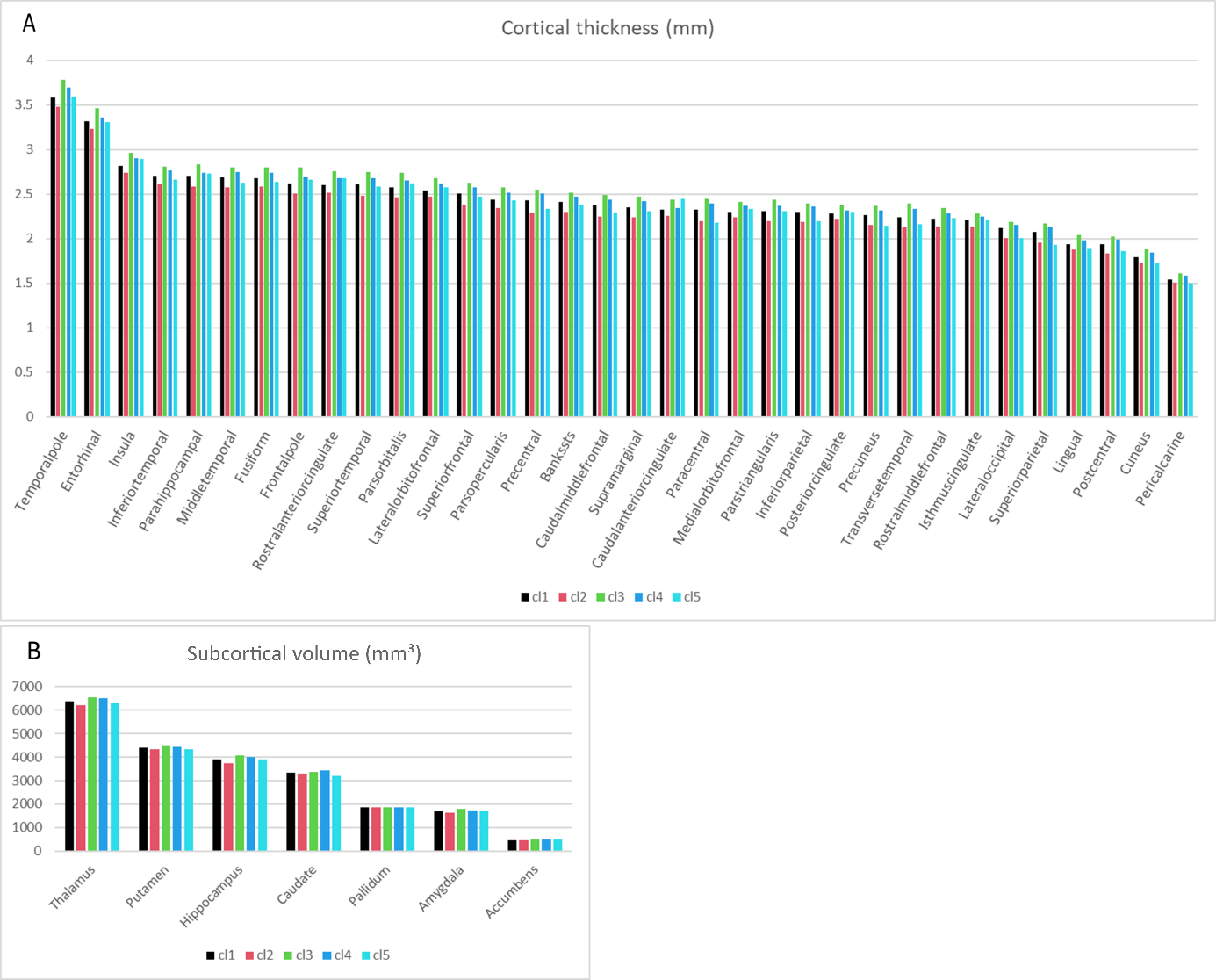


**Supplementary Table 1**. Test–retest reliability of FreeSurfer 7 measures before and after scanner upgrade in two human phantoms.

| **FreeSurfer 7** | **Phantom 01** | | **Phantom 02** | |
| --- | --- | --- | --- | --- |
| **Measure (mm or mm^3^)** | **abs diff** | **% change** | **abs diffss** | **% change** |
| Bankssts (mm) | 0.04 | -1.32 | 0.01 | 0.33 |
| caudalanteriorcingulate | 0.04 | 1.72 | 0.02 | -1.06 |
| caudalmiddlefrontal | 0.03 | -1.27 | 0 | -0.14 |
| cuneus | 0.01 | -0.56 | 0.03 | -1.35 |
| entorhinal | 0.05 | -1.63 | 0.01 | 0.20 |
| fusiform | 0.05 | -1.73 | 0.03 | 1.20 |
| inferiorparietal | 0.03 | -1.06 | 0.05 | -2.01 |
| inferiortemporal | 0.03 | -1.13 | 0.03 | 1.09 |
| isthmuscingulate | 0.02 | -0.82 | 0.02 | 0.87 |
| lateraloccipital | 0 | 0.07 | 0.01 | -0.44 |
| lateralorbitofrontal | 0.15 | -5.34 | 0.05 | -1.89 |
| lingual | 0.01 | 0.37 | 0.04 | 2.07 |
| medialorbitofrontal | 0.08 | -3.22 | 0.09 | -3.84 |
| middletemporal | 0.03 | 0.97 | 0.03 | 0.92 |
| parahippocampal | 0.04 | 1.35 | 0.07 | 2.34 |
| paracentral | 0 | -0.13 | 0.01 | 0.27 |
| parsopercularis | 0.03 | -1.17 | 0.03 | -1.08 |
| parsorbitalis | 0.14 | -4.60 | 0.09 | -3.22 |
| parstriangularis | 0.12 | -4.27 | 0.09 | -3.33 |
| pericalcarine | 0.06 | 2.88 | 0.05 | 2.65 |
| postcentral | 0.04 | -1.69 | 0.02 | -1.09 |
| posteriorcingulate | 0 | 0.21 | 0.01 | 0.36 |
| precentral | 0.04 | -1.61 | 0 | -0.06 |
| precuneus | 0.05 | -2.13 | 0.05 | -2.01 |
| rostralanteriorcingulate | 0.03 | -1.09 | 0.02 | -0.74 |
| rostralmiddlefrontal | 0.11 | -4.24 | 0.12 | -5.04 |
| superiorfrontal | 0.03 | -1.08 | 0.05 | -1.70 |
| superiorparietal | 0.05 | -2.29 | 0.05 | -2.34 |
| superiortemporal | 0.03 | -1.15 | 0.01 | -0.36 |
| supramarginal | 0.05 | -2.01 | 0.01 | 0.20 |
| frontalpole | 0.08 | -2.74 | 0.09 | -3.32 |
| temporalpole | 0.05 | -1.32 | 0.02 | -0.53 |
| transversetemporal | 0.09 | 3.35 | 0 | 0.20 |
| insula | 0.02 | -0.59 | 0.07 | 2.37 |
| Thalamus (mm^3^) | 66.10 | 0.80 | 46.75 | 0.70 |
| Caudate | 28.10 | 0.61 | 62.80 | -1.41 |
| Putamen | 82.75 | 1.53 | 34.00 | 0.68 |
| Pallidum | 6.75 | -0.34 | 6.95 | 0.39 |
| Hippocampus | 19.25 | 0.43 | 24.40 | 0.66 |
| Amygdala | 38.40 | -2.16 | 10.75 | 0.58 |
| Accumbens.area | 37.65 | 5.56 | 17.15 | -3.01 |
| Mean across regions |  | -0.80 |  | -0.53 |

*Note*: Phantom 01 and Phantom 02 were each scanned before and after the upgrade from a 3.0 T Philips Achieva to a 3.0 T Philips Achieva dStream system, using the same imaging protocol. Absolute and percentage differences in cortical thickness (mm) and subcortical volumes (mm³) are shown. Mean percentage change across regions and phantoms = −0.67%, consistent with previously reported within-scanner test–retest variability < 1% in phantoms.

**Supplementary Table 2**. Baseline CSF biomarkers of Alzheimer’s disease in a sub-sample of 286 participants from the Gothenburg H70– Birth Cohort 1944 by cluster.

|  | **Cluster 1 (N=103)** | **Cluster 2 (N=59)** | **Cluster 3 (N=45)** | **Cluster 4 (N=60)** | **Cluster 5 (N=19)** | **Total (N=286)** | **p *** |
| --- | --- | --- | --- | --- | --- | --- | --- |
| **Aβ 1-42 positive** | 50 (49.0) | 24 (42.1) | 18 (40.0) | 29 (49.2) | 10 (52.6) | 131 (46.5) | 0.750 |
| **Total tau positive** | 30 (29.1) | 14 (23.7) | 14 (31.1) | 22 (36.7) | 11 (57.9) | 91 (31.8) | 0.204 |
| **p-tau 181 positive** | 6 (5.8) | 2 (3.4) | 1 (2.2) | 5 (8.3) | 2 (10.5) | 16 (5.6) | 0.750 |

*Note.* Data are presented as numbers (percentage) for categorical variables. Pathological cut-off values for biomarker positivity were defined as follows: β-amyloid 42 (Aβ42) ≤ 530 pg/mL, total tau (t-tau) ≥ 350 pg/mL, and phosphorylated tau at threonine 181 (p-tau181) ≥ 80 pg/mL.

Abbreviations: CSF, cerebrospinal fluid.

Missing data: Amyloid-β 1-42 (n=4).

* FDR-corrected p value <0.05.

**Supplementary Table 3.** Comparison of baseline characteristics between participants with and without CSF data in the Gothenburg H70– Birth Cohort 1944.

|  | **CSF subsample (N=286)** | **CSF not available (N=460)** | **Total (N=746)** | **p *** |
| --- | --- | --- | --- | --- |
| **Sociodemographic and genetic factors** | | | | |
| Sex |  |  |  | 0.231 |
| Men | 147 (51.4) | 206 (44.8) | 353 (47.3) |  |
| Women | 139 (48.6) | 254 (55.2) | 393 (52.7) |  |
| Education, years |  |  |  | 0.189 |
| Primary school | 33 (11.5) | 49 (10.7) | 82 (11.0) |  |
| Secondary school | 159 (55.6) | 209 (45.5) | 368 (49.4) |  |
| Higher education | 94 (32.9) | 201 (43.8) | 295 (39.6) |  |
| APOE ε4 (one/two) | 102 (36.2) | 138 (30.8) | 240 (32.9) | 0.307 |
| MMSE score | 28.98 ± 1.23 | 29.16 ± 1.11 | 29.09 ± 1.16 | **0.047 *** |
| **Cardiovascular risk factors** | | | | |
| Current/former smoking | 177 (62.1) | 279 (60.9) | 456 (61.4) | 0.811 |
| At-risk alcohol consumption | 83 (29.1) | 152 (33.1) | 235 (31.6) | 0.490 |
| Physical inactivity | 8 (2.8) | 17 (3.8) | 25 (3.4) | 0.626 |
| Overweight/obese (BMI ≥25 kg/m^2^) | 143 (52.0) | 257 (59.6) | 400 (56.7) | 0.192 |
| Elevated triglycerides | 94 (32.9) | 179 (39.2) | 273 (36.7) | 0.231 |
| Reduced HDL cholesterol (mmol/L) | 27 (9.4) | 59 (12.9) | 86 (11.6) | 0.307 |
| LDL cholesterol (mmol/L) |  |  |  | 0.857 |
| T1 (≤3) | 95 (33.2) | 160 (35.1) | 255 (34.4) |  |
| T2 (3.1-3.9) | 99 (34.6) | 156 (34.2) | 255 (34.4) |  |
| T3 (≥4) | 92 (32.2) | 140 (30.7) | 232 (31.3) |  |
| Hypertension (≥140/90 mmHg) | 210 (73.4) | 309 (67.3) | 519 (69.7) | 0.231 |
| **Medical conditions** | | | | |
| Heart disease | 40 (14.0) | 92 (20.0) | 132 (17.7) | 0.189 |
| Stroke/TIA | 14 (4.9) | 43 (9.3) | 57 (7.6) | 0.189 |
| Diabetes |  |  |  | 0.626 |
| Normoglycemia | 124 (43.4) | 189 (41.2) | 313 (42.0) |  |
| Prediabetes | 126 (44.1) | 197 (42.9) | 323 (43.4) |  |
| Diabetes | 36 (12.6) | 73 (15.9) | 109 (14.6) |  |
| Depression (major/minor) | 26 (9.1) | 33 (7.2) | 59 (7.9) | 0.571 |
| Traumatic brain injuries | 99 (34.6) | 143 (31.1) | 242 (32.4) | 0.566 |
| **Blood biomarkers** | | | | |
| Homocysteine > 13 µmol/L | 104 (36.6) | 167 (37.4) | 271 (37.1) | 0.856 |
| C-reactive protein ≥ 8 mg/L | 20 (7.1) | 36 (8.1) | 56 (7.7) | 0.745 |
| **Neuroimaging biomarkers** |  |  |  |  |
| DTI’s Average FA |  |  |  | 0.626 |
| T1 (<0.3) | 85 (31.4) | 152 (34.6) | 237 (33.4) |  |
| T2 (0.3 - 0.4) | 89 (32.8) | 148 (33.7) | 237 (33.4) |  |
| T3 (>0.4) | 97 (35.8) | 139 (31.7) | 236 (33.2) |  |
| WMHV (mm^3^) |  |  |  | 0.626 |
| T1 (<2.7) | 97 (34.0) | 151 (33.0) | 248 (33.4) |  |
| T2 (2.7 – 5.5) | 101 (35.4) | 147 (32.1) | 248 (33.4) |  |
| T3 (>5.5) | 87 (30.5) | 160 (34.9) | 247 (33.2) |  |
| Cerebral microbleeds | 25 (8.8) | 56 (12.3) | 81 (10.9) | 0.307 |
| Lacunes (3-15 mm) | 27 (9.5) | 25 (5.5) | 52 (7.0) | 0.189 |
| Large infarctions | 5 (1.8) | 10 (2.2) | 15 (2.0) | 0.785 |
| Enlarged PVS CS |  |  |  | 0.189 |
| 0-10 | 63 (22.2) | 133 (29.1) | 196 (26.5) |  |
| $\geq$ 11 | 221 (77.8) | 324 (70.9) | 545 (73.5) |  |
| Enlarged PVS BG |  |  |  | 0.626 |
| 0-10 | 219 (77.1) | 364 (79.6) | 583 (78.7) |  |
| $\geq$ 11 | 65 (22.9) | 93 (20.4) | 158 (21.3) |  |

*Note.* Data are presented as Mean ± Standard deviations for continuous variables or number (percentage) for categorical variables. Abbreviations: APOE-ɛ4, apolipoprotein E gene-ɛ4 allele; MMSE, Mini-Mental State Examination; BMI, body mass index; HDL, High-density lipoprotein; LDL, Low-density lipoprotein; TIA, transient ischemic attack; MRI, Magnetic Resonance Imaging; AD, Alzheimer’s Disease; DTI, diffusion tensor imaging; FA, fractional anisotropy; WMHV, white matter hyperintensities volume; PVS, perivascular spaces.

Missing data: Education (n=1), APOE-ɛ4 (n=16), MMSE (n=4), Smoking (n=3), At-risk alcohol consumption (n=2), Physical activity (n=15), BMI (n=2), Elevated triglycerides (n=3), Reduced HDL cholesterol (n=4), LDL cholesterol (n=4), Hypertension (n=1), Heart disease (n=1), Prediabetes/diabetes (n=1), Depression (n=2), FA (n=36), Homocysteine (n=16), C-reactive protein (n=16), WMHV (n=3), Cerebral microbleeds (n=6), Lacunes (n=3), Large infarctions (n=4), Enlarged PVS Centrum semiovale (n=5), Enlarged PVS Basal ganglia (n=5).
*FDR-corrected p value <0.05.

**Supplementary Table 4**. Odds ratios (ORs) and 95% confidence intervals (CIs) from four multinomial regression models (clinical, neuroimaging, inflammation, and CSF), comparing baseline clusters with Cluster 1 as the reference.

|  | **Cluster 2** | | | | **Cluster 3** | | | | **Cluster4** | | | | **Cluster 5** | | | |
| --- | --- | --- | --- | --- | --- | --- | --- | --- | --- | --- | --- | --- | --- | --- | --- | --- |
|  | **OR** | | **95% CI** | | **OR** | | **95% CI** | | **OR** | | **95% CI** | | **OR** | | **95% CI** | |
| **Clinical model** | | | | | | | | | | | | | | | | |
| Sex |  |  | |  |  |  | |  |  |  | |  |  |  | |  |
| Men | Reference | | | | Reference | | | | Reference | | | | Reference | | | |
| Women | 1.13 | 0.70 | | 1.83 | 1.16 | 0.71 | | 1.88 | 1.20 | 0.76 | | 1.90 | 1.50 | 0.74 | | 3.03 |
| Education |  |  | |  |  |  | |  |  |  | |  |  |  | |  |
| Primary/lower secondary | Reference | | | | Reference | | | | Reference | | | | Reference | | | |
| Higher secondary | 1.27 | 0.64 | | 2.54 | 2.06 | 0.84 | | 5.04 | 2.14 | 0.94 | | 4.86 | 0.63 | 0.23 | | 1.73 |
| Higher education | 0.93 | 0.44 | | 1.99 | 2.13 | 0.84 | | 5.38 | **2.52*** | **1.08** | | **5.87** | 1.09 | 0.39 | | 3.02 |
| APOE ε |  |  | |  |  |  | |  |  |  | |  |  |  | |  |
| No ε allele | Reference | | | | Reference | | | | Reference | | | | Reference | | | |
| One/two ε allele | 1.19 | 0.74 | | 1.92 | 0.95 | 0.57 | | 1.57 | 1.16 | 0.73 | | 1.84 | 1.19 | 0.59 | | 2.40 |
| Current/former smoking | 1.08 | 0.67 | | 1.74 | 0.86 | 0.54 | | 1.38 | **0.62*** | **0.40** | | **0.95** | 0.79 | 0.40 | | 1.56 |
| At-risk alcohol consumption | **1.83*** | **1.13** | | **2.97** | 1.12 | 0.67 | | 1.86 | 0.93 | 0.57 | | 1.53 | **2.09*** | **1.05** | | **4.16** |
| Physical inactivity | 0.62 | 0.20 | | 1.99 | 0.55 | 0.11 | | 2.66 | 0.48 | 0.10 | | 2.35 | 1.82 | 0.43 | | 7.63 |
| Overweight/obese (BMI ≥25 kg/m2) | 1.09 | 0.67 | | 1.79 | **0.57*** | **0.35** | | **0.94** | 1.04 | 0.65 | | 1.66 | 0.89 | 0.43 | | 1.83 |
| Elevated triglycerides | 1.06 | 0.63 | | 1.79 | 0.91 | 0.52 | | 1.57 | **0.55*** | **0.32** | | **0.95** | 0.46 | 0.20 | | 1.06 |
| Reduced HDL cholesterol (mmol/L) | 0.90 | 0.44 | | 1.83 | 0.82 | 0.36 | | 1.89 | 1.20 | 0.59 | | 2.43 | 0.97 | 0.32 | | 2.91 |
| LDL cholesterol (mmol/L) |  |  | |  |  |  | |  |  |  | |  |  |  | |  |
| T1 (≤3) | Reference | | | | Reference | | | | Reference | | | | Reference | | | |
| T2 (3.1-3.9) | 0.89 | 0.50 | | 1.56 | 0.71 | 0.39 | | 1.29 | 1.19 | 0.68 | | 2.08 | 1.05 | 0.43 | | 2.59 |
| T3 (≥4) | 0.73 | 0.40 | | 1.31 | 0.83 | 0.46 | | 1.49 | 0.99 | 0.55 | | 1.76 | 1.80 | 0.75 | | 4.30 |
| Hypertension (≥140/90 mmHg) | 1.10 | 0.65 | | 1.89 | 1.02 | 0.61 | | 1.70 | 1.00 | 0.62 | | 1.62 | 1.04 | 0.49 | | 2.18 |
| Heart disease | 0.77 | 0.40 | | 1.49 | 1.27 | 0.65 | | 2.48 | 1.70 | 0.92 | | 3.15 | **3.44*** | **1.48** | | **8.01** |
| Stroke/TIA | 1.18 | 0.53 | | 2.62 | 0.94 | 0.36 | | 2.46 | 0.93 | 0.38 | | 2.24 | 1.20 | 0.35 | | 4.09 |
| Diabetes |  |  | |  |  |  | |  |  |  | |  |  |  | |  |
| Normoglycemia | Reference | | | | Reference | | | | Reference | | | | Reference | | | |
| Prediabetes | 1.00 | 0.59 | | 1.68 | 1.13 | 0.68 | | 1.88 | 0.91 | 0.56 | | 1.47 | 1.25 | 0.60 | | 2.63 |
| Diabetes | **2.54*** | **1.27** | | **5.06** | 0.85 | 0.36 | | 2.02 | 1.06 | 0.49 | | 2.28 | 1.50 | 0.48 | | 4.72 |
| Depression (major/minor) | 0.67 | 0.31 | | 1.45 | 0.77 | 0.35 | | 1.69 | **0.17*** | **0.05** | | **0.56** | 0.93 | 0.32 | | 2.66 |
| Traumatic brain injuries | 1.27 | 0.79 | | 2.04 | 1.04 | 0.63 | | 1.71 | 1.34 | 0.85 | | 2.13 | 0.86 | 0.41 | | 1.78 |
| **Neuroimaging model** | | | | | | | | | | | | | | | | |
| DTI’s Average FA |  |  | |  |  |  | |  |  |  | |  |  |  | |  |
| T1 (<0.3) | Reference | | | | Reference | | | | Reference | | | | Reference | | | |
| T2 (0.3 - 0.4) | 0.58 | 0.34 | | 1.01 | 1.20 | 0.65 | | 2.25 | 1.62 | 0.93 | | 2.81 | 0.96 | 0.44 | | 2.08 |
| T3 (>0.4) | **0.46** | **0.25** | | **0.83** | 1.59 | 0.85 | | 2.85 | 1.43 | 0.80 | | 2.48 | 0.53 | 0.21 | | 1.24 |
| WMHV, mm3 |  |  | |  |  |  | |  |  |  | |  |  |  | |  |
| T1 (<2.7) | Reference | | | | Reference | | | | Reference | | | | Reference | | | |
| T2 (2.7 – 5.5) | 0.78 | 0.44 | | 1.41 | **0.45*** | **0.26** | | **0.77** | **0.51*** | **0.31** | | **0.84** | 0.72 | 0.35 | | 1.47 |
| T3 (>5.5) | 1.28 | 0.70 | | 2.34 | **0.50*** | **0.27** | | **0.92** | 0.67 | 0.38 | | 1.16 | **0.23*** | **0.08** | | **0.66** |
| Cerebral microbleeds (presence) | 0.68 | 0.32 | | 1.29 | 0.96 | 0.47 | | 1.94 | 0.75 | 0.36 | | 1.47 | 0.15 | 0.02 | | 1.16 |
| Lacunes 3-15 mm | **0.35** | **0.14** | | **0.85** | 0.54 | 0.20 | | 1.35 | **0.18** | **0.05** | | **0.61** | 0.62 | 0.17 | | 2.37 |
| Large infarctions | 4.91 | 0.97 | | 25.83 | 2.06 | 0.17 | | 23.73 | 1.52 | 0.12 | | 17.10 | **17.44** | **1.94** | | **156.13** |
| Enlarged PVS in centrum semiovale |  |  | |  |  |  | |  |  |  | |  |  |  | |  |
| 0-10 | Reference | | | | Reference | | | | Reference | | | | Reference | | | |
| ≥11 | 1.23 | 0.71 | | 2.09 | 1.38 | 0.80 | | 2.32 | 1.18 | 0.72 | | 1.90 | 1.96 | 0.88 | | 4.46 |
| Enlarged PVS in basal ganglia |  |  | |  |  |  | |  |  |  | |  |  |  | |  |
| 0-10 | Reference | | | | Reference | | | | Reference | | | | Reference | | | |
| ≥11 | 1.52 | 0.86 | | 2.57 | 1.02 | 0.54 | | 1.92 | 1.37 | 0.77 | | 2.35 | 1.32 | 0.57 | | 3.09 |
| **Inflammation model** | | | | | | | | | | | | | | | | |
| Homocysteine > 13 µmol/L | 1.01 | 0.66 | | 1.52 | 0.76 | 0.48 | | 1.19 | 0.82 | 0.55 | | 1.24 | **0.40*** | **0.19** | | **0.84** |
| C-reactive protein ≥ 8 mg/L | 1.69 | 0.83 | | 3.47 | 1.32 | 0.59 | | 2.95 | 0.68 | 0.28 | | 1.66 | 2.02 | 0.76 | | 5.37 |
| **CSF model** | | | | | | | | | | | | | | | | |
| Amyloid-β 1-42 ≥ 530 pg/mL | 0.76 | 0.39 | | 1.45 | 0.69 | 0.34 | | 1.41 | 1.01 | 0.53 | | 1.91 | 1.16 | 0.43 | | 3.08 |
| Total tau ≥ 350 pg/mL | 0.76 | 0.36 | | 1.58 | 1.10 | 0.51 | | 2.35 | 1.41 | 0.72 | | 2.77 | **3.35*** | **1.22** | | **9.14** |
| Phosphorylated tau ≥ 80 pg/mL | 0.57 | 0.11 | | 2.91 | 0.37 | 0.04 | | 3.14 | 1.47 | 0.43 | | 5.04 | 1.90 | 0.35 | | 10.2 |

*Note.* CSF analyses were performed in a subset of 286 participants.

Abbreviations: APOE-ɛ4, apolipoprotein E gene-ɛ4 allele; BMI, body mass index; HDL, High-density lipoprotein; LDL, Low-density lipoprotein; TIA, transient ischemic attack; DTI, diffusion tensor imaging; FA, fractional anisotropy; WMHV, white matter hyperintensities volume; PVS, perivascular spaces; CSF, cerebrospinal fluid.

Asterisks (*) indicate statistical significance (p value <0.05).

**Supplementary Table 5.** β-coefficients and standard errors (SE) from Generalized Linear Models (GLM) for the associations between clusters of gray matter and six cognitive outcomes (global cognition, episodic memory, attention and perceptual speed, executive function, verbal fluency, visuospatial abilities) all 746 subjects at baseline.

| ***Cluster 2*** | | | | |  |
| --- | --- | --- | --- | --- | --- |
| **Cognitive Domain** | **β (Estimate)** | **SE** | **t value** | **Unadjusted p *** | **FDR adjusted p *** |
| Global cognition | -0.12 | 0.06 | -1.94 | 0.052 | 0.085 |
| Episodic memory | -0.19 | 0.08 | -2.47 | **0.014 *** | 0.082 |
| Attention/Speed | -0.12 | 0.08 | -1.48 | 0.140 | 0.168 |
| Executive function | -0.16 | 0.08 | -1.91 | 0.057 | 0.085 |
| Verbal fluency | 0.02 | 0.09 | 0.19 | 0.849 | 0.849 |
| Visuospatial abilities | -0.21 | 0.10 | -2.02 | **0.044 *** | 0.085 |
| ***Cluster 3*** | | | | |  |
| **Cognitive Domain** | **β (Estimate)** | **SE** | **t value** | **Unadjusted p *** | **FDR adjusted p *** |
| Global cognition | 0.08 | 0.07 | 1.18 | 0.239 | 0.593 |
| Episodic memory | 0.07 | 0.08 | 0.85 | 0.395 | 0.593 |
| Attention/Speed | 0.08 | 0.09 | 0.90 | 0.367 | 0.593 |
| Executive function | 0.01 | 0.09 | 0.06 | 0.956 | 0.956 |
| Verbal fluency | 0.16 | 0.09 | 1.71 | 0.087 | 0.522 |
| Visuospatial abilities | 0.05 | 0.11 | 0.48 | 0.631 | 0.757 |
| ***Cluster 4*** | | | | |  |
| **Cognitive Domain** | **β (Estimate)** | **SE** | **t value** | **Unadjusted p *** | **FDR adjusted p *** |
| Global cognition | 0.16 | 0.06 | 2.58 | **0.010 *** | **0.031 *** |
| Episodic memory | 0.16 | 0.08 | 2.06 | **0.040 *** | 0.060 |
| Attention/Speed | 0.08 | 0.08 | 0.97 | 0.331 | 0.397 |
| Executive function | 0.07 | 0.08 | 0.85 | 0.398 | 0.398 |
| Verbal fluency | 0.24 | 0.09 | 2.77 | **0.006 **** | **0.031 *** |
| Visuospatial abilities | 0.22 | 0.10 | 2.23 | **0.026 *** | 0.052 |
| ***Cluster 5*** | | | | |  |
| **Cognitive Domain** | **β (Estimate)** | **SE** | **t value** | **Unadjusted p *** | **FDR adjusted p *** |
| Global cognition | -0.04 | 0.10 | -0.37 | 0.709 | 0.851 |
| Episodic memory | -0.01 | 0.12 | -0.10 | 0.924 | 0.924 |
| Attention/Speed | -0.07 | 0.12 | -0.57 | 0.572 | 0.851 |
| Executive function | -0.12 | 0.13 | -0.98 | 0.329 | 0.851 |
| Verbal fluency | 0.10 | 0.13 | 0.72 | 0.474 | 0.851 |
| Visuospatial abilities | -0.07 | 0.16 | -0.44 | 0.663 | 0.851 |

*Note*: Generalized Linear Models (GLM). Cluster 1 is set as reference for prediction measures.

Asterisks (*) indicate statistical significance (p<0.05).

**Supplementary Table 6.** Model summaries from sex- and education-adjusted linear mixed-effects models predicting **(A)** cognitive subdomains, **(B)** MMSE, **(C)** WMHV.

| **A) Verbal fluency** | **β** | **SE** | **p *** |
| --- | --- | --- | --- |
| **Intercept** | -1.22 | 0.14 | 0.000 |
| **Time** | -0.20 | 0.05 | 0.000 |
| **Cluster 2** | 0.12 | 0.11 | 0.486 |
| **Cluster 3** | 0.17 | 0.11 | 0.217 |
| **Cluster 4** | 0.30 | 0.10 | 0.011 |
| **Cluster 5** | 0.25 | 0.18 | 0.331 |
| **Time × Cluster 2** | -0.23 | 0.09 | **0.040 *** |
| **Time × Cluster 3** | -0.12 | 0.08 | 0.732 |
| **Time × Cluster 4** | -0.16 | 0.08 | 0.178 |
| **Time × Cluster 5** | -0.24 | 0.13 | 0.340 |
| **B) MMSE** | **β** | **SE** | **p *** |
| **Intercept** | 28.26 | 0.18 | 0.000 |
| **Time** | -0.32 | 0.12 | 0.008 |
| **Cluster 2** | 0.01 | 0.15 | 0.952 |
| **Cluster 3** | 0.21 | 0.15 | 0.158 |
| **Cluster 4** | -0.02 | 0.14 | 0.868 |
| **Cluster 5** | 0.29 | 0.23 | 0.206 |
| **Time × Cluster 2** | -0.45 | 0.21 | **0.035 *** |
| **Time × Cluster 3** | -0.05 | 0.21 | 0.816 |
| **Time × Cluster 4** | 0.33 | 0.20 | 0.094 |
| **Time × Cluster 5** | -0.32 | 0.34 | 0.347 |
| **C) WMHV** | **β** | **SE** | **p *** |
| **Intercept** | 5.43 | 1.12 | <0.001 |
| **Time** | 2.95 | 0.36 | <0.001 |
| **Cluster 2** | 2.84 | 0.93 | 0.002 |
| **Cluster 3** | –1.01 | 0.91 | 0.267 |
| **Cluster 4** | –1.69 | 0.84 | 0.045 |
| **Cluster 5** | –2.94 | 1.44 | 0.042 |
| **Time × Cluster 2** | 1.84 | 0.64 | **0.004 *** |
| **Time × Cluster 3** | –0.42 | 0.62 | 0.498 |
| **Time × Cluster 4** | –1.00 | 0.57 | 0.078 |
| **Time × Cluster 5** | –1.01 | 0.97 | 0.298 |

Note: Cluster 1 serves as the reference category for all cluster comparisons. Estimates (β-coefficients and standard errors [SE]) reflect fixed effects for intercepts (ref = Cluster 1) and interactions with time (baseline vs 5-years follow-up).

* FDR-corrected p value <0.05.

**Supplementary Table 7.** Baseline sociodemographic, clinical, biochemical, neuroimaging characteristics, and GM clusters by survival status over follow-up (participants, dropouts, deceased).

|  | **Follow-up (N=436)** | **Deceased (N=12)** | **Dropout (N=298)** | **p *** |
| --- | --- | --- | --- | --- |
| **Sociodemographic and genetic factors** | | | | |
| Sex |  |  |  | 0.499 |
| Men | 203 (46.6%) | 8 (66.7%) | 142 (47.7%) |  |
| Women | 233 (53.4%) | 4 (33.3%) | 156 (52.3%) |  |
| Education, years |  |  |  | 0.065 |
| Primary school | 36 (8.3%) | 2 (16.7%) | 44 (14.8%) |  |
| Secondary school | 210 (48.3%) | 9 (75.0%) | 149 (50.0%) |  |
| Higher education | 189 (43.4%) | 1 (8.3%) | 105 (35.2%) |  |
| APOE ε4 (one/two) | 136 (31.4%) | 2 (16.7%) | 102 (35.8%) | 0.397 |
| MMSE score | 29.2 ± 1.1 | 29.4 ± 0.8 | 29.0 ± 1.2 | 0.091 |
| **Cardiovascular risk factors** | | | | |
| Current/former smoking | 244 (56.1%) | 7 (58.3%) | 205 (69.3%) | **0.041 *** |
| Alcohol risk consumption | 135 (31.0%) | 4 (33.3%) | 96 (32.3%) | 0.963 |
| Physical inactivity | 9 (2.1%) | 1 (8.3%) | 15 (5.1%) | 0.133 |
| Overweight/obese (BMI ≥25 kg/m^2^) | 218 (50.1%) | 9 (75.0%) | 173 (58.2%) | 0.103 |
| Elevated triglycerides | 142 (32.7%) | 6 (50.0%) | 125 (42.1%) | 0.098 |
| Reduced HDL cholesterol (mmol/L) | 38 (8.8%) | 1 (8.3%) | 47 (15.9%) | 0.098 |
| LDL cholesterol (mmol/L) |  |  |  | 0.468 |
| T1 (≤3) | 141 (32.5%) | 2 (16.7%) | 112 (37.8%) |  |
| T2 (3.1-3.9) | 157 (36.2%) | 6 (50.0%) | 92 (31.1%) |  |
| T3 (≥4) | 136 (31.3%) | 4 (33.3%) | 92 (31.1%) |  |
| Hypertension (≥140/90 mmHg) | 290 (66.7%) | 8 (66.7%) | 221 (74.2%) | 0.186 |
| **Medical conditions** | | | | |
| Heart disease | 69 (15.9%) | 3 (25.0%) | 60 (20.1%) | 0.430 |
| Stroke/TIA | 29 (6.7%) | 1 (8.3%) | 27 (9.1%) | 0.564 |
| Diabetes status |  |  |  | 0.103 |
| Normoglycemia | 199 (45.7%) | 2 (16.7%) | 112 (37.6%) |  |
| Prediabetes | 182 (41.8%) | 6 (50.0%) | 135 (45.3%) |  |
| Diabetes | 54 (12.4%) | 4 (33.3%) | 51 (17.1%) |  |
| Depression (major/minor) | 29 (6.7%) | 1 (8.3%) | 29 (9.7%) | 0.468 |
| Traumatic brain injuries | 150 (34.4%) | 4 (33.3%) | 88 (29.5%) | 0.499 |
| **Blood biomarkers** | | | | |
| Homocysteine > 13 µmol/L | 149 (34.7%) | 8 (66.7%) | 114 (39.6%) | 0.113 |
| C-reactive protein ≥ 8 mg/L | 25 (5.8%) | 2 (16.7%) | 29 (10.1%) | 0.124 |
| **Neuroimaging biomarkers** |  |  |  |  |
| DTI’s Average FA |  |  |  | 0.113 |
| T1 (<0.3) | 125 (29.7%) | 5 (45.5%) | 107 (38.5%) |  |
| T2 (0.3 - 0.4) | 139 (33.0%) | 4 (36.4%) | 94 (33.8%) |  |
| T3 (>0.4) | 157 (37.3%) | 2 (18.2%) | 77 (27.7%) |  |
| WMHV (mm^3^) |  |  |  | 0.098 |
| T1 (<2.7) | 162 (37.2%) | 3 (25.0%) | 83 (28.0%) |  |
| T2 (2.7 – 5.5) | 148 (34.0%) | 3 (25.0%) | 97 (32.8%) |  |
| T3 (>5.5) | 125 (28.7%) | 6 (50.0%) | 116 (39.2%) |  |
| Cerebral microbleeds | 43 (9.9%) | 2 (16.7%) | 36 (12.2%) | 0.564 |
| Lacunes (3-15 mm) | 30 (6.9%) | 2 (16.7%) | 20 (6.8%) | 0.514 |
| Large infarctions | 4 (0.9%) | 1 (8.3%) | 10 (3.4%) | 0.098 |
| Enlarged PVS CS |  |  |  | 0.993 |
| 0-10 | 115 (26.4%) | 3 (25.0%) | 78 (26.5%) |  |
| ≥ 11 | 320 (73.6%) | 9 (75.0%) | 216 (73.5%) |  |
| Enlarged PVS BG |  |  |  | 0.963 |
| 0-10 | 344 (79.1%) | 9 (75.0%) | 230 (78.2%) |  |
| ≥ 11 | 91 (20.9%) | 3 (25.0%) | 64 (21.8%) |  |
| **Clusters** |  |  |  | 0.222 |
| 1 (n=278) | 156 (35.8%) | 4 (33.3%) | 118 (39.6%) |  |
| 2 (n=142) | 76 (17.4%) | 5 (41.7%) | 61 (20.5%) |  |
| 3 (n=121) | 78 (17.9%) | 2 (16.7%) | 41 (13.8%) |  |
| 4 (n=157) | 102 (23.4%) | 0 (0.0%) | 55 (18.5%) |  |
| 5 (n=48) | 24 (5.5%) | 1 (8.3%) | 23 (7.7%) |  |

Data are presented as Mean ± Standard deviations for continuous variables or number (percentage) for categorical variables.

Abbreviations: *APOE*-ɛ4, apolipoprotein E gene-ɛ4 allele; MMSE, Mini-Mental State Examination; BMI, body mass index; HDL, High-density lipoprotein; LDL, Low-density lipoprotein; T, tertile; TIA, transient ischemic attack; DTI, diffusion tensor imaging; FA, fractional anisotropy; WMHV, white matter hyperintensity volume; PVS, perivascular spaces; CS, centrum semiovale; BG, basal ganglia.

Missing data: Education (n=1), *APOE*-ɛ4 (n=16), MMSE (n=4), Smoking (n=3), Alcohol consumption (n=2), Physical activity (n=15), BMI (n=2), Elevated triglycerides (n=3), Reduced HDL cholesterol (n=4), LDL cholesterol (n=4), Hypertension (n=1), Heart disease (n=1), Prediabetes/diabetes (n=1), Depression (n=2), Homocysteine (n=16), C-reactive protein (n=16), Fractional anisotropy (n=36), WMHV (n=3), Cerebral microbleeds (n=6), Lacunes (n=3), Large infarctions (n=4), Enlarged PVS Centrum semiovale (n=5), Enlarged PVS Basal ganglia (n=5).

*FDR-corrected p value <0.05.
